# Supplementary material for: Integrated cohort of esophageal squamous cell cancer reveals genomic features underlying clinical characteristics
Source: Nat Commun. 2022 Sep 7;13:5268. doi: 10.1038/s41467-022-32962-1 (PMC9452532; doi:10.1038/s41467-022-32962-1)
Supplement: Supplementary file 1 — Supplementary Information [file 41467_2022_32962_MOESM1_ESM.pdf]

Supplementary Figure 1

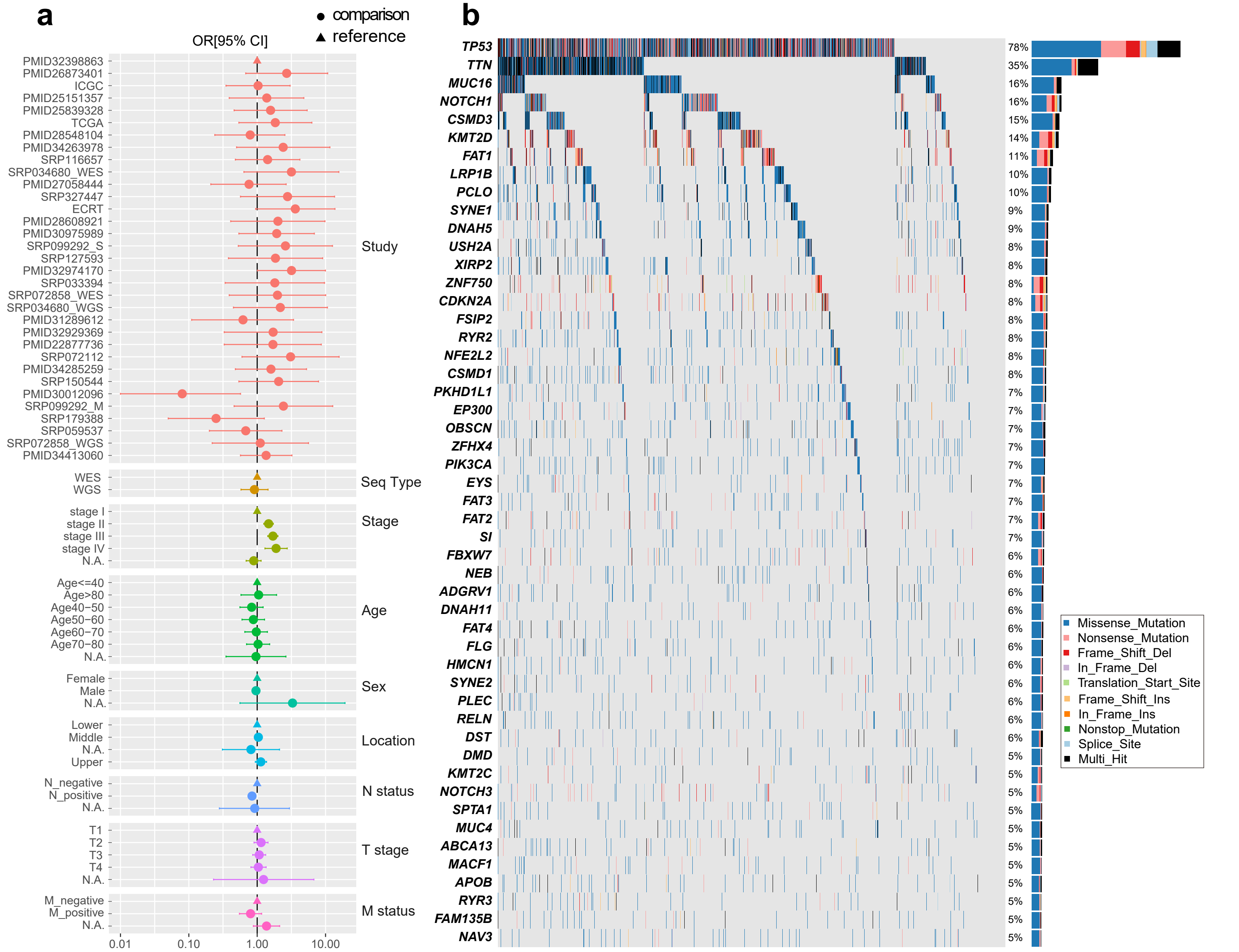

a, the multivariable linear regression of the non-silent mutational load (as the dependent variable) in ESCC-META cohort (n=1930). The box and arms in each line of the forest plot represent the odd ratio (OR) value and 95% confidence intervals.

b, the oncoplot of the top 50 common mutated genes in ESCC-META cohort.

Source data are provided as a Source Data file.

Supplementary Figure 2

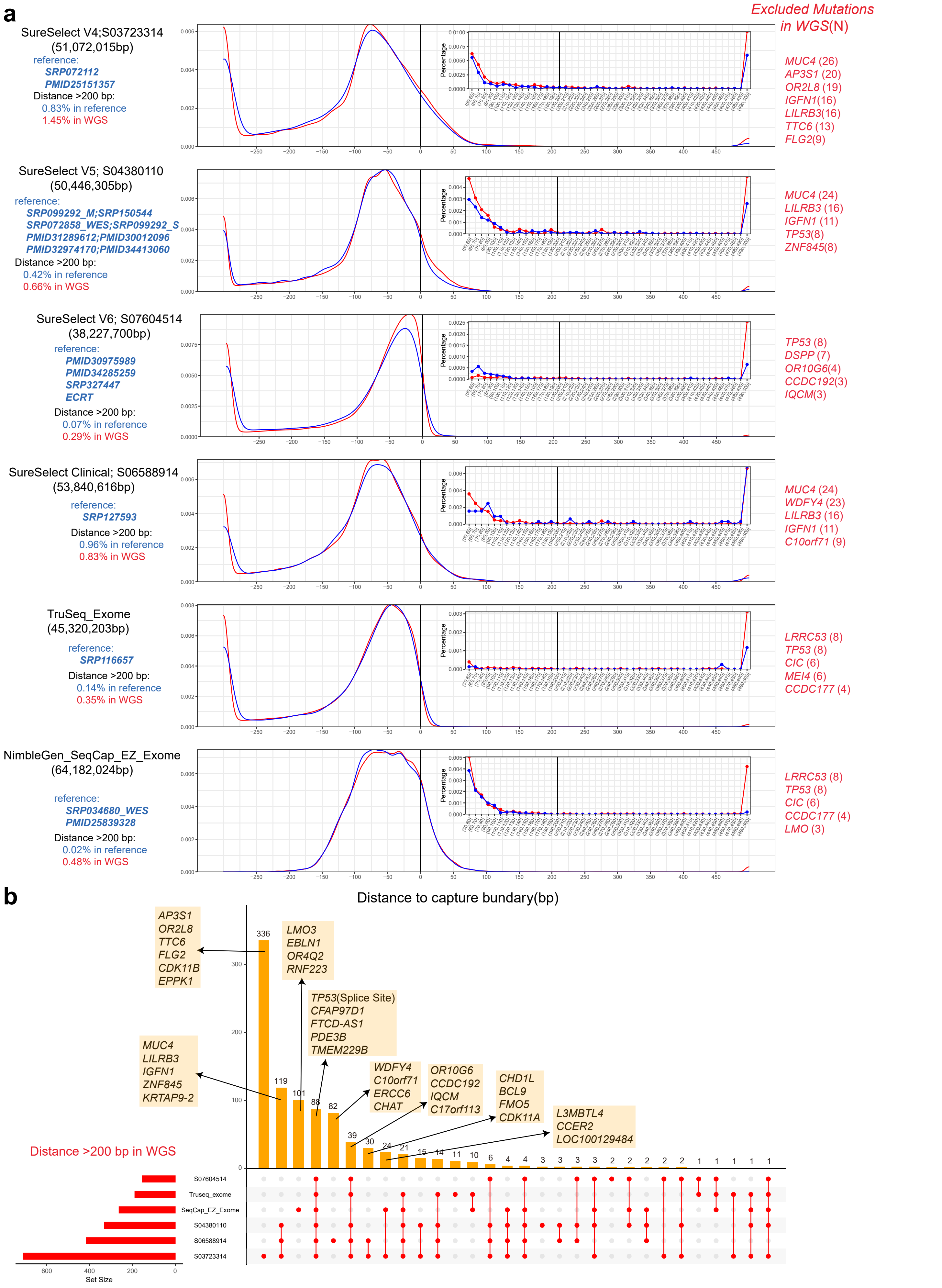

a, the distributions of the distance in reference set (blue) and testing set (red). For each non-silent SNV, the distance was defined as its locus to the nearest capture boundary. The positive value represented out of capture range of the mutational site, and the negative value represented within capture range.
b, the upset plot of the excluded mutational sites among the six capture platforms. The mainly involved genes were labelled in the orange boxes.
Source data are provided as a Source Data file.

Supplementary Figure 3

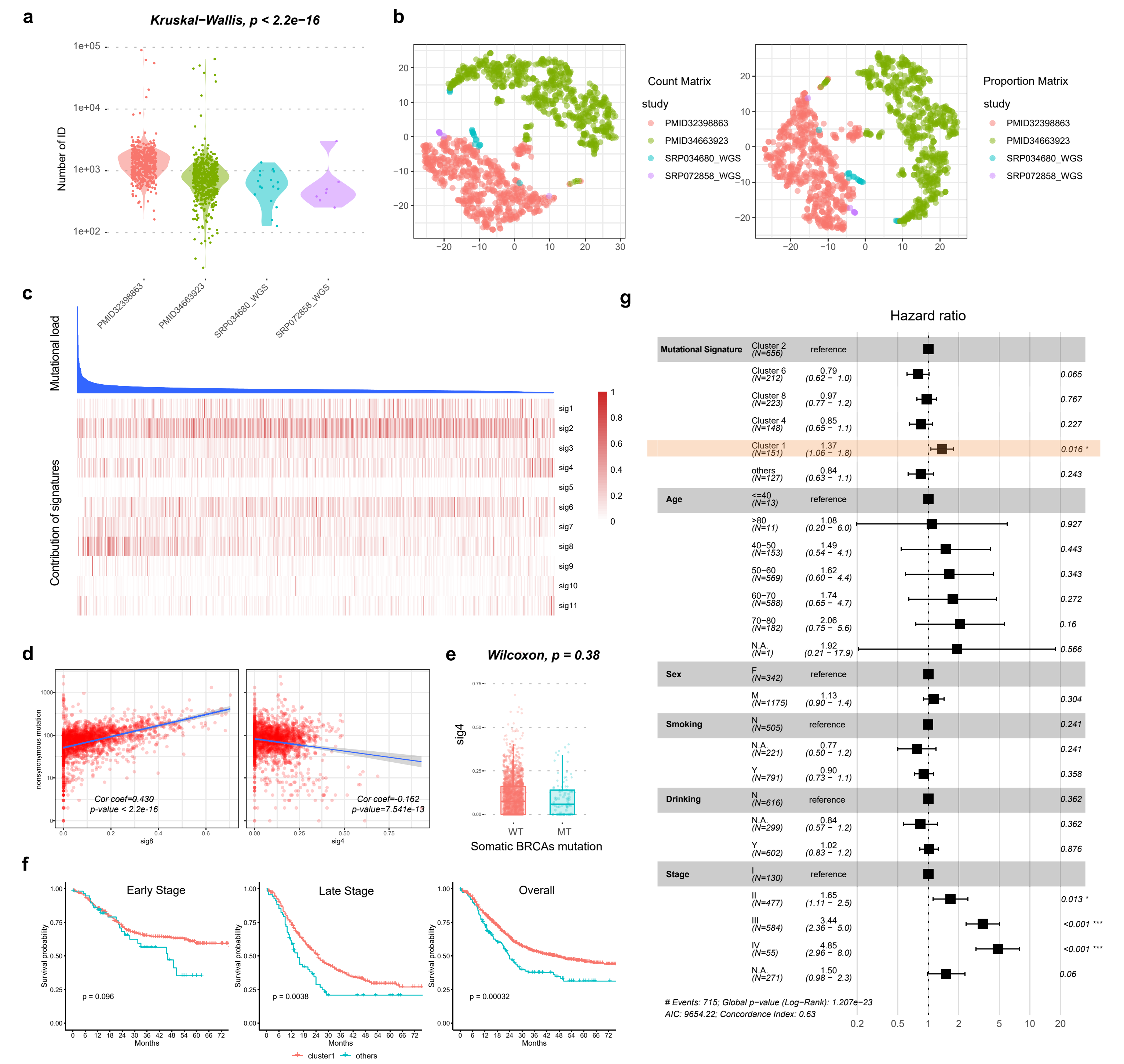

a, the distribution of total number of somatic IDs in the WGS genomes among the four datasets. The two-side Kruskal-Wallis test was used to detect the difference among datasets.
b, the t-SNE analysis of the count matrix (left) or proportion matrix (right) of the 83 ID types in all WGS samples (n=1084). The dots were colored by the dataset.
c, the heatmap of the contribution of identified 11 signatures in ESCC-MEAT cohort. The genomes were ranked by mutational load from left to right.
d, the scatter plots between the contributions of sig8 (left) or sig4 (right) and mutational load. The Pearson's correlation coefficient and its significance test were used to measure the correlation. The blue line and the grey band represent the fitted regression line and 95% confidence intervals.
e, the box plot of sig4 contributions between BRCAs mutational status. The two-side Wilcox test was used to measure the significance. In the boxplots, the lower extreme line, lower end of box, inner line of box, upper end of box and upper extreme line represent the value of (Q1-1.5*IQR), Q1, Q2, Q3 and (Q3+1.5*IQR) respectively. Q1, 25th quartile; Q2, 50th quartile or the median value; Q3, 75th quartile. The interquartile range (IQR) is distance between Q1 and Q3 (Q3-Q1).
f, the overall survival curve in early (n=607) or late-stage (n=639) patients, comparing the cluster1 patients and others. The two-side log rank test was used to detect the significance.
g, the forest plot of multivariable Cox regression results. The box and arms in each line represents the HR value and 95% confidence intervals.
Source data are provided as a Source Data file.

Supplementary Figure 4

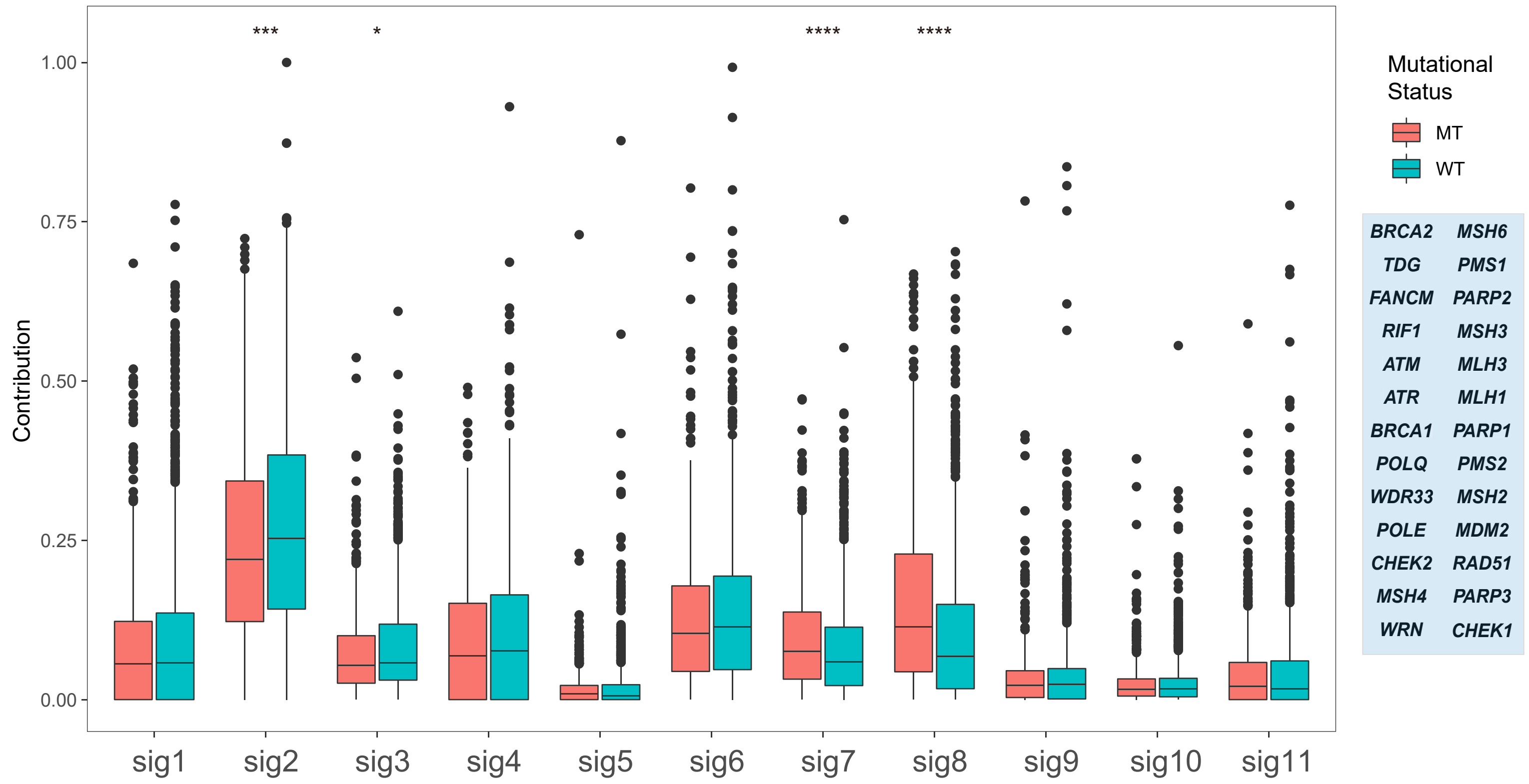

Comparison of the contribution of mutational signatures between tumors with or without mutations in DNA-repair pathway (genes listed in the right box). The p-value of two-side Wilcox test was used to measure the significance, * indicating p<0.05, ** p<0.01, *** p<0.001, **** p<0.0001. In the boxplots, the lower extreme line, lower end of box, inner line of box, upper end of box and upper extreme line represent the value of (Q1-1.5*IQR), Q1, Q2, Q3 and (Q3+1.5*IQR) respectively. Q1, 25th quartile; Q2, 50th quartile or the median value; Q3, 75th quartile. The interquartile range (IQR) is distance between Q1 and Q3 (Q3-Q1).
Source data are provided as a Source Data file.

Supplementary Figure 5

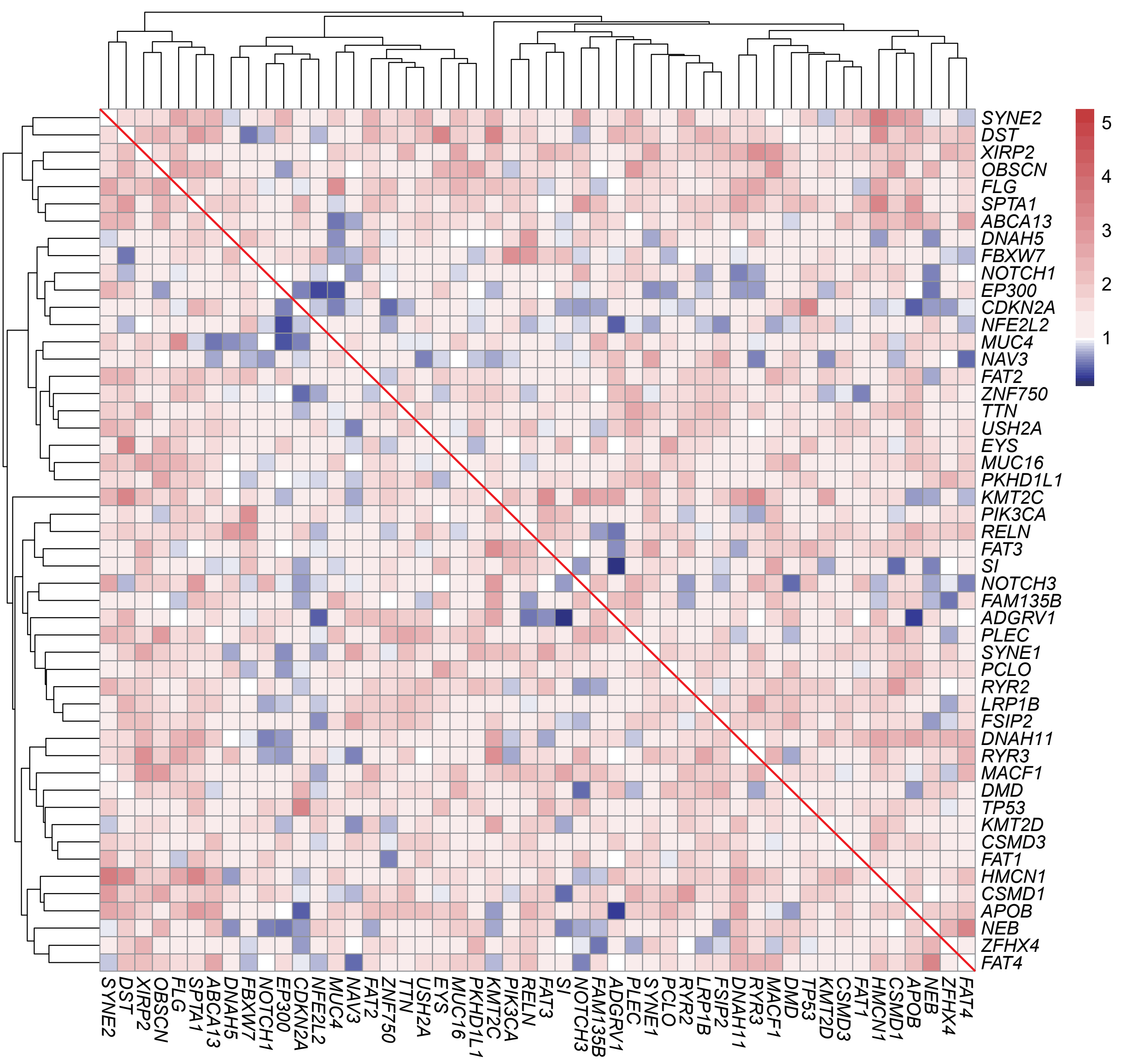

The heatmap of pairwise interactions of top 50 genes, which was indicated by the odds ratio (OR) value of the co-occurrent events. The OR>1 (red color) indicating co-occurrence, OR<1 (blue color) indicating mutually exclusive.
Source data are provided as a Source Data file.

Supplementary Figure 6

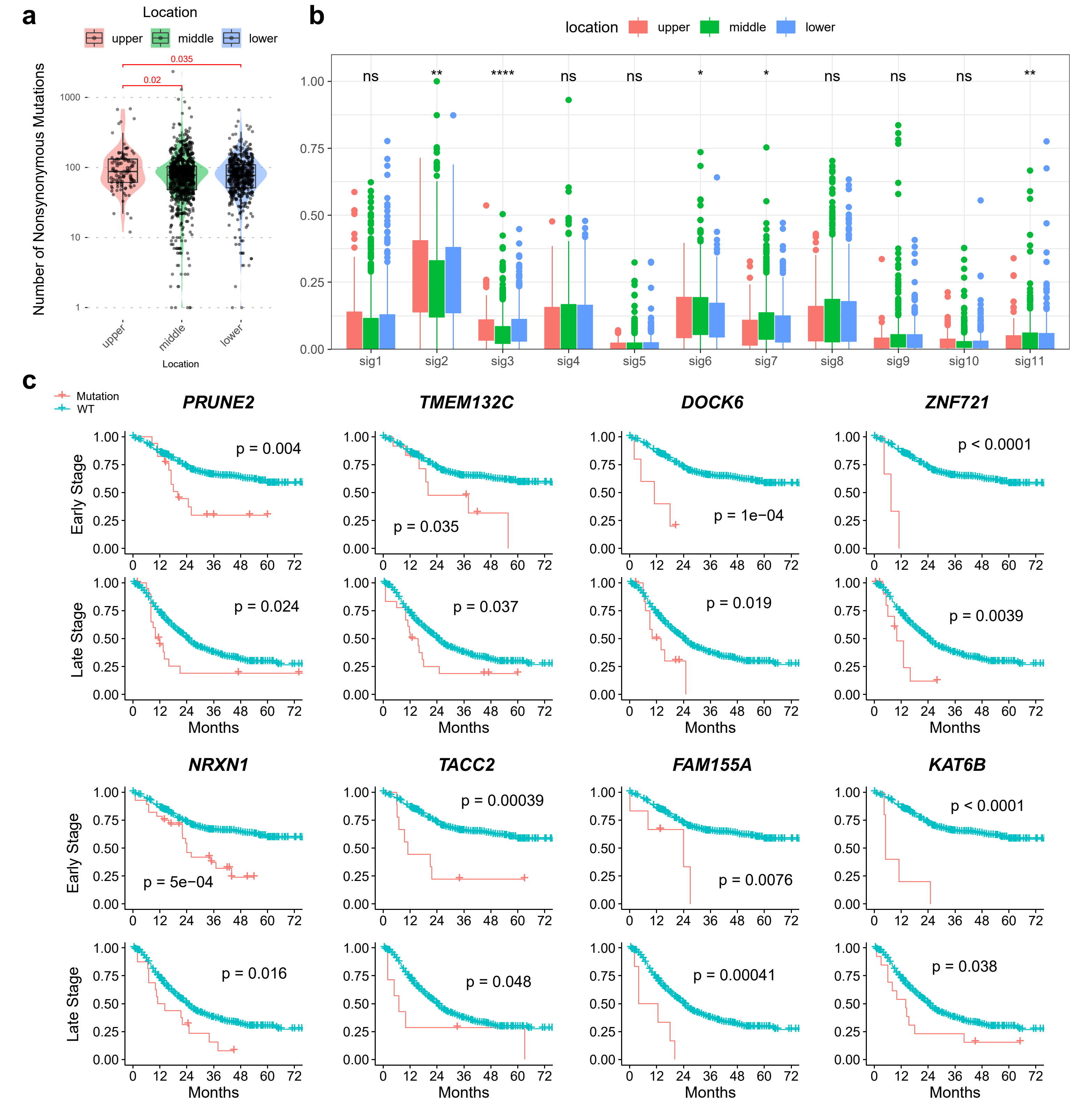

a, comparison of mutational load among different tumor locations. The p-value of two-side Wilcox test was used to measure the significance.
b, comparison of contribution of the 11 signatures among different tumor locations. The Kruskal-Wallis test was used to detect the significance, ns indicating p≥0.05, * indicating p<0.05, ** p<0.01, *** p<0.001, **** p<0.0001. In the boxplots of a and b, the lower extreme line, lower end of box, inner line of box, upper end of box and upper extreme line represent the value of (Q1-1.5*IQR), Q1, Q2, Q3 and (Q3+1.5*IQR) respectively. Q1, 25th quartile; Q2, 50th quartile or the median value; Q3, 75th quartile. The interquartile range (IQR) is distance between Q1 and Q3 (Q3-Q1). c, survival plots of some significant genes in early or late-stage patients of ESCC-META. The two-side log rank test was used to detect the significance.
Source data are provided as a Source Data file.

Supplementary Figure 7

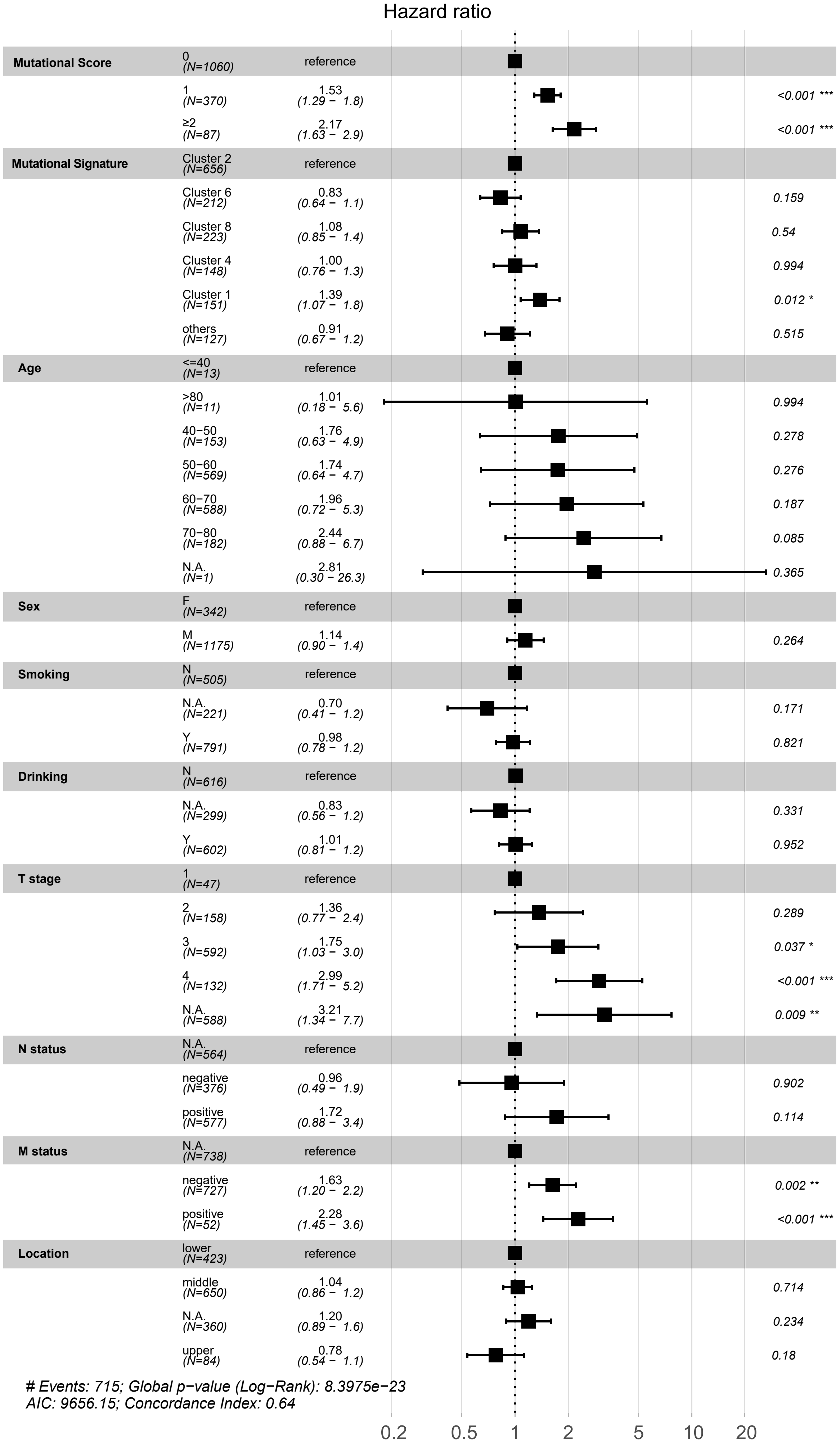

The results of multivariable Cox regression of overall survival to the mutational score and clinical factors in ESCC-META cohort. The box and arms in each line represents the HR value and 95% confidence intervals.
Source data are provided as a Source Data file.

Supplementary Table 1, the 15 datasets re-analyzed from raw reads data

Dataset	Passed SNVs	Smples	Taget methods
SRP116657	29422	78	TruSeqExome Enrichment kit
SRP034680_WES	27842	71	NimbleGenEZ 44M
SRP327447	20870	46	Agilent SureSelect Human All Exon V6
ECRT	30166	42	Agilent SureSelect Human All Exon V6
SRP099292_S	11312	36	Agilent SureSelect Human All Exon V5
SRP127593	10161	32	SureSelectXT Clinical Research Exome panel
SRP033394	3599	19	SureSelect Human All Exon 50M
SRP072858_WES	8610	18	Agilent SureSelect Human All ExonV5
SRP034680_WGS	185989	17	WGS
SRP072112	5205	11	Agilent SureSelect Human All Exon V4
SRP150544	4712	10	Agilent SureSelect Human All Exon V5
SRP099292_M	3388	9	SureSelect V5 whole exon
SRP059537	4051	9	N.A.
SRP179388	6555	8	N.A.
SRP072858_WGS	53988	7	WGS

Supplementary Table 2, the collection of druggable genes

**Mutated Gene**	**FDA Approved Drug**
BRCA1	olaparib
BRCA2	olaparib
EGFR	mobocertinib
VEGFA	bevacizumab
ROS1	crizotinib;entrectinib
ALK	crizotinib;ceritinib
BRAF	vemurafenib; dabrafenib
KRAS	sotorasib
NTRK1	larotrectinib
MET	capmatinib
CD274	atezolizumab; avelumab
RET	selpercatinib
ERBB2	afatinib
FGFR1	erdafitinib

Supplementary Table 3, list of the 22 genes significantly mutated genes

Gene	Tx	Total mutations	CDS length(bp)	Mutational frequence	Mutsig Q value	OncodriveCLUST clusterScores	Mutational Density	dN/dS
TP53	NM_000546	1595	1179	78.19%	2.98E-13	0.4815	7009.54	76.90
NOTCH1	NM_017617	291	7665	15.54%	4.71E-14	0.3648	196.71	14.95
KMT2D	NM_003482	287	16611	14.15%	5.22E-14	0.2618	89.52	7.24
ZNF750	NM_024702	171	2169	8.24%	4.42E-13	0.3927	408.49	20.88
CDKN2A	NM_000077	150	468	8.19%	0	0.2766	1660.69	143.00
NFE2L2	NM_006164	152	1815	7.62%	3.13E-13	0.4492	433.92	21.14
EP300	NM_001429	132	7242	7.15%	0	0.5401	94.44	13.00
PIK3CA	NM_006218	139	3204	6.94%	1.09E-13	0.7301	224.78	27.60
FBXW7	NM_018315	123	1881	6.48%	0	0.4108	338.81	30.00
NOTCH3	NM_000435	98	6963	5.49%	4.61E-13	0.2114	72.92	5.11
CREBBP	NM_001079846	93	7212	4.92%	0	0.2774	66.81	7.00
AJUBA	NM_032876	74	1614	4.09%	0	0.3968	237.56	36.50
RB1	NM_000321	62	2784	3.63%	0	0.2375	115.39	12.00
PPFIA2	NM_001220473	64	3741	3.37%	6.22E-09	0.2351	88.64	6.78
KRT5	NM_000424	60	1770	2.95%	0	0.6267	175.64	9.83
KEAP1	NM_012289	55	1872	2.75%	0	0.6175	152.23	17.33
CASP8	NM_001080125	49	1614	2.54%	0	0.4025	157.30	16.33
CUL3	NM_001257197	41	2106	2.49%	0	0.2206	100.87	19.50
TGFBR2	NM_003242	47	1701	2.49%	0	0.3202	143.16	6.71
ZBBX	NM_001199201	38	2517	2.23%	0	0.2142	78.22	6.33
ATP13A5	NM_198505	42	3654	2.12%	0	0.2063	59.56	6.67
IRF2BPL	NM_024496	43	2388	2.02%	1.19E-05	0.7992	93.30	14.33
