# Supplementary material for: Integrated cohort of esophageal squamous cell cancer reveals genomic features underlying clinical characteristics
Source: Nat Commun. 2022 Sep 7;13:5268. doi: 10.1038/s41467-022-32962-1 (PMC9452532; doi:10.1038/s41467-022-32962-1)
Supplement: Supplementary file 3 — Description of Additional Supplementary Files [file 41467_2022_32962_MOESM3_ESM.pdf]

Supplementary Data 1, the 18 datasets prepared from published mutational list

Supplementary Data 2, all included patients in the ESCC-META cohort

Supplementary Data 3, incomplete covered regions by one or some capture platforms
The coverage ratio for each region were in each in capture platform were shown in colored boxes. We did not add the flank regions of capture range in this calculation.

Supplementary Data 4, the 96 SBS profile of WGS patients (n=1084)

Supplementary Data 5, the 96 SBS features of 11 identified mutational signatures

Supplementary Data 6, cosine similarity between the 11 identified mutational signatures and known COSMIC signature

Supplementary Data 7, the 1888 genes of mutational frequency more than 1% and the corresponding parameters to estimate mutated significance

Supplementary Data 8, results of mutational interaction analysis

Supplementary Data 9, results of age and tumor location related comparison

Supplementary Data 10, results of multivariable Cox analysis in discovery set, which is used in building the mutational score
