# Supplementary material for: Integrated cohort of esophageal squamous cell cancer reveals genomic features underlying clinical characteristics
Source: Nat Commun. 2022 Sep 7;13:5268. doi: 10.1038/s41467-022-32962-1 (PMC9452532; doi:10.1038/s41467-022-32962-1)
Supplement: Supplementary file 14 — Reporting Summary [file 41467_2022_32962_MOESM14_ESM.pdf]

nature portfolio

Corresponding author(s): Baosheng Li

Last updated by author(s): Jul 26, 2022

Reporting Summary

Nature Portfolio wishes to improve the reproducibility of the work that we publish. This form provides structure for consistency and transparency in reporting. For further information on Nature Portfolio policies, see our Editorial Policies and the Editorial Policy Checklist.

Statistics

For all statistical analyses, confirm that the following items are present in the figure legend, table legend, main text, or Methods section.

n/a	Confirmed	
☐	☒	The exact sample size (*n*) for each experimental group/condition, given as a discrete number and unit of measurement
☐	☒	A statement on whether measurements were taken from distinct samples or whether the same sample was measured repeatedly
☐	☒	The statistical test(s) used AND whether they are one- or two-sided<br>*Only common tests should be described solely by name; describe more complex techniques in the Methods section.*
☐	☒	A description of all covariates tested
☐	☒	A description of any assumptions or corrections, such as tests of normality and adjustment for multiple comparisons
☐	☒	A full description of the statistical parameters including central tendency (e.g. means) or other basic estimates (e.g. regression coefficient) AND variation (e.g. standard deviation) or associated estimates of uncertainty (e.g. confidence intervals)
☐	☒	For null hypothesis testing, the test statistic (e.g. *F*, *t*, *r*) with confidence intervals, effect sizes, degrees of freedom and *P* value noted<br>*Give P values as exact values whenever suitable.*
☒	☐	For Bayesian analysis, information on the choice of priors and Markov chain Monte Carlo settings
☒	☐	For hierarchical and complex designs, identification of the appropriate level for tests and full reporting of outcomes
☒	☐	Estimates of effect sizes (e.g. Cohen's *d*, Pearson's *r*), indicating how they were calculated

Our web collection on statistics for biologists contains articles on many of the points above.

Software and code

Policy information about availability of computer code

Data collection	No software used.
Data analysis	The used softwares in data process include SRA-Tools (v2.11),fastp (v0.23),BWA (v0.7.1), GATK (v4.1), Mutect2, CrossMap(v0.2.6), ANNOVAR (December 2019 version) and BAM-matcher. The statistical analysis and visualizations were all performed in R (4.1.0) with the help of packages of survival (3.2), survminer (0.4.9), meta (4.9), maftools (2.8), Rtsne (0.15), NMF (0.23.0), mutSignatures (2.1.1), dplyr (1.0.6) and ggplot2 (3.3.5).

For manuscripts utilizing custom algorithms or software that are central to the research but not yet described in published literature, software must be made available to editors and reviewers. We strongly encourage code deposition in a community repository (e.g. GitHub). See the Nature Portfolio guidelines for submitting code & software for further information.

Data

Policy information about availability of data

All manuscripts must include a data availability statement. This statement should provide the following information, where applicable:

- Accession codes, unique identifiers, or web links for publicly available datasets
- A description of any restrictions on data availability
- For clinical datasets or third party data, please ensure that the statement adheres to our policy

The raw WES data of ECRT generated in this study have been deposited in National Genomics Data Center (NGDC) of China National Center for Bioinformation under the accession code of HRA002596. The integrated ESCC-META dataset is available at synapse under the accession code of syn27304838. The relevant results data generated in this study are provided in the Supplementary Information. Source data are provided with this paper.

The other raw sequence data used in this study are available in the SRA database under accession code of SRP099292, SRP033394, SRP059537, SRP150544, SRP072112, SRP179388, SRP072858, SRP127593, SRP327447, SRP034680 and SRP116657.

The mutational records in coding region of the integrated ESCC-META datasets are public available in syn27304838, while the SNVs in non-coding region and the full clinical information would be provided on reasonable request.

Field-specific reporting

Please select the one below that is the best fit for your research. If you are not sure, read the appropriate sections before making your selection.

- [x] Life sciences
- [ ] Behavioural & social sciences
- [ ] Ecological, evolutionary & environmental sciences

For a reference copy of the document with all sections, see nature.com/documents/nr-reporting-summary-flat.pdf

Life sciences study design

All studies must disclose on these points even when the disclosure is negative.

Sample size	No sample size calculation was performed. We had collected all the possible ESCC genome data from public source and the final sample size of 1930 patients, which was the largest size we could obtain at present.
Data exclusions	Target sequence other than WES and the low coverage WGS data (mean coverage< 10) for CNV analysis were not included in ESCC-META. If the multiple tumor samples were collected from different time point, only the earliest tumor sample (at diagnosis or before any treatment) were used. We excluded patients with multiple primary tumors or esophageal tumor of unclear pathological diagnosis. The samples apart from primary tumor tissue, such as from metastatic sites, were also excluded.
Replication	We verified the established mutational score in an independent testing set of ECRT. In the testing set, the eight gene mutational score was validated as a reliable prognostic model in ESCC for clinical application.
Randomization	This work was based on retrospective data and the potential covariates of clinical variables were adjusted by multivariate regression analysis.
Blinding	In data preparing, the two authors indecently processed and checked the raw data. In the primary genomic data process, the authors were blinded to group allocation(such as the clinical information). In the validation of the mutational score, the test set was processed and used by authors (Yan Yi and Qianrong Wang) who were blind to the group allocation.

Reporting for specific materials, systems and methods

We require information from authors about some types of materials, experimental systems and methods used in many studies. Here, indicate whether each material, system or method listed is relevant to your study. If you are not sure if a list item applies to your research, read the appropriate section before selecting a response.

Materials & experimental systems

n/a	Involved in the study	
x		Antibodies
x		Eukaryotic cell lines
x		Palaeontology and archaeology
x		Animals and other organisms
	x	Human research participants
	x	Clinical data
x		Dual use research of concern

Methods

n/a	Involved in the study	
x		ChIP-seq
x		Flow cytometry
x		MRI-based neuroimaging

Human research participants

Policy information about studies involving human research participants

Population characteristics	The most patients in the ESCC-META dataset were involved from public available resources. The ECRT patients(n=42) were involved in a multicenter, randomized phase III clinical trial of ChiCTR-IPR-15007172. The patients were all diagnosed locally advanced ESCC tumor and received radical concurrent chemoradiotherapy as the first tumor treatment. The mean age of the 42 patients is 63 years, and there are 13 female patients and 29 male patients involved.
Recruitment	The recruitment stage of clinical trail was from 2015 to 2020 and patients were recruited from 31 centers and all the initial evaluations and assignment works were centralized in Shandong Tumor Hospital.
Ethics oversight	The study was approved by the ethics committee of Shandong Tumor Hospital.

Note that full information on the approval of the study protocol must also be provided in the manuscript.

Clinical data

Policy information about clinical studies

All manuscripts should comply with the ICMJEguidelines for publication of clinical research and a completedCONSORT checklist must be included with all submissions.

Clinical trial registration	ChiCTR-IPR-15007172
Study protocol	The full study protocol is available in the platform of Chinese Clinical Trial Registry
Data collection	The general information of the participates and the diagnostic of the tumor was obtained in the involvement of the clinical trails. The survival data was collected under the plan of follow-up of the clinical trial, in which the patients would be contacted by telephone, e-mail, or clinic visit, every 3 months after last radiotherapy for up to 2 years subjects and then every 6 months for 3-5 years.
Outcomes	The primary endpoint was overall survival (OS), which is defined as the period from randomization to death from any cause.
